# Atrial Mitral and Tricuspid Regurgitation: Sex Matters. A Call for Action to Unravel the Differences Between Women and Men

**DOI:** 10.3389/fcvm.2022.877592

**Published:** 2022-06-13

**Authors:** Francisco Gual-Capllonch, José Ignacio Sáenz de Ibarra, Antoni Bayés-Genís, Victoria Delgado

**Affiliations:** ^1^Heart Institute, Clínica Rotger, Palma, Spain; ^2^Cardiac Surgery Department, Clínica Rotger, Palma, Spain; ^3^Heart Institute, Hospital Universitari Germans Trias i Pujol, Barcelona, Spain; ^4^Department of Medicine, Universitat Autònoma de Barcelona, Barcelona, Spain; ^5^CIBERCV, Instituto de Salud Carlos III, Madrid, Spain

**Keywords:** female sex, atrial mitral regurgitation, atrial tricuspid regurgitation, heart failure, atrial fibrillation

## Abstract

Atrial functional regurgitation is caused by atrioventricular annulus dilation, with normal leaflets and ventricular dimensions and function within the normal range. Its occurrence, in both mitral and tricuspid valves, implies a worse prognosis due to the hemodynamic derangement they produce, but also constitutes a marker of greater comorbidity and more advanced disease. Predisposing conditions for these heart valve dysfunctions are mainly atrial fibrillation and heart failure with preserved ejection fraction. However, other factors like female sex also may be involved and influence their incidence, especially for atrial tricuspid regurgitation. In the present review, we analyze sex differences in the reported prevalence of atrial mitral and tricuspid regurgitation, and suggest possible mechanisms involved. Finally, we underline potential therapeutic and preventive strategies to reduce the burden of these heart valve disorders and discuss research gaps.

Secondary regurgitation of atrioventricular valves is characterized by geometric changes of the valvular apparatus secondary to dilation and/or dysfunction of the ventricles or atria. In contrast to primary atrioventricular regurgitation, the leaflets of the mitral and tricuspid valves are structurally normal. Accordingly, the management of secondary atrioventricular valve regurgitation differs significantly from that of primary regurgitation since it needs to target the underlying mechanism first rather than fixing directly the anatomy and competence of the atrioventricular valve (1). Secondary atrioventricular valve regurgitation due to dilation and dysfunction of the left or right ventricles are associated with an excess of mortality (2–5). Additionally, it is increasingly recognized a specific type of secondary atrioventricular regurgitation caused by mitral or tricuspid valve annulus dilation but with left and right ventricular dimensions and function within the normal range. This type of secondary atrioventricular valve regurgitation is known as atrial mitral regurgitation (AMR) (6) and atrial tricuspid regurgitation (ATR) (7), and is characterized by normal leaflet motion -type I of the Carpentier classification- and a diminished coaptation surface caused by atrial and subsequent atrioventricular annulus valve dilation. The characteristics of the patients with significant AMR and ATR have been described in a few cohort-based studies (6, 7). Patients are characterized for being elderly, having a high prevalence of atrial fibrillation (AF) (8, 9) and heart failure with preserved ejection fraction (HFpEF) (10, 11). Both AF and HFpEF are associated with atrial cardiomyopathy and/or atrial failure (12) and increased pressure overload of the atria leading to dilation and dysfunction of these cardiac chambers (13). Furthermore, the volume overload imposed by atrioventricular regurgitation may aggravate the atrial and atrioventricular valvular annulus dilation, leading to more advanced atrial myopathy and impairment of atrioventricular valve regurgitation. The excess of mortality and heart failure complications associated to the presence of significant AMR and ATR has been demonstrated in patients with AF and HFpEF (14–17).

While it is well known that women with AF and/or HFpEF (which are associated with AMR and ATR) present at a more advanced course of the disease as compared to men and the implementation of the available therapies may differ between the two sexes (18, 19), the sex differences in AMR and ATR have not been extensively investigated. In the present review article we provide an overview of the sex differences in the prevalence and pathophysiology of AMR and ATR and discuss the potential gaps in knowledge from the diagnostic and therapeutic point of view that need further research in order to improve the outcomes of men and women (Figure 1).

**Figure 1:**
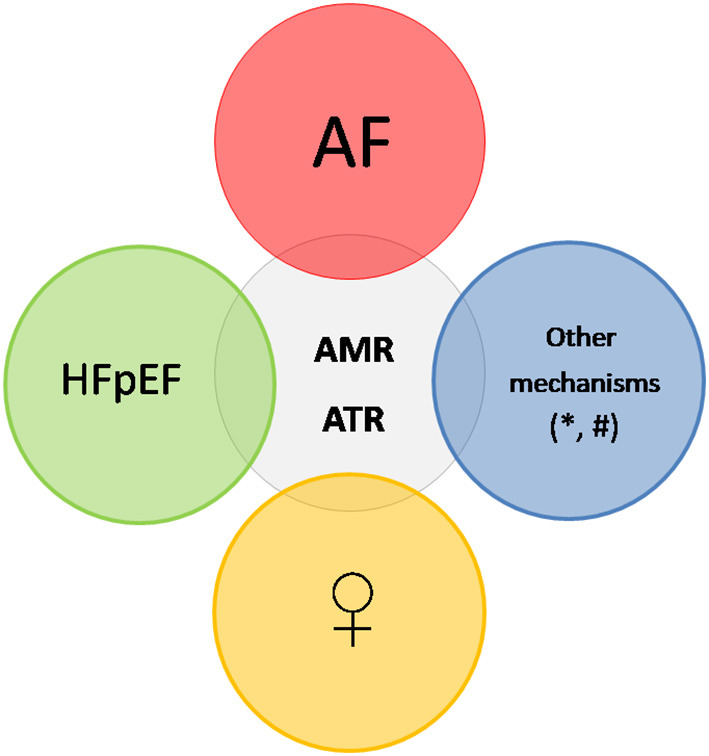
Differents factors contribute to the occurrence of AMR and ATR. The most frequent substrate is AF, which leads to atrial and atrioventricular annulus dilation. HFpEF also elicits AMR and ATR, due to left and right atrial dilation as a result of pressure overload, and also constitutes a frequent trigger for AF. ^*^denotes other variables or mechanisms, which may contribute to the occurrence of AMR, like hypervolemia, hamstringing of the posterior mitral leaflet or insufficient leaflet growth. # denotes other variables or mechanisms, which may contribute to the occurrence of ATR, like older age, hypervolemia, pulmonary hypertension, intracardiac lead or insufficient leaflet growth. Prevalence data and suggested pathophysiologic mechanisms indicate that female patients are at higher risk for these heart valve disorders. AMR, atrial mitral regurgitation; ATR, atrial tricuspid regurgitation; AF, atrial fibrillation; HFpEF, heart failure with preserved ejection fraction; PH, pulmonary hypertension.

## Differences in Prevalence of AMR Between Women and Men

Women have a distinct etiologic spectrum of mitral valve disease compared to men, with a higher prevalence of mitral valve prolapse and rheumatic mitral valve regurgitation and lower prevalence of ischemic mitral regurgitation (20, 21). However, differences in prevalence of AMR between sexes is not clearly established, since the definition of AMR is relatively new and not consistent across the various observational studies and there are potential selection biases inherent to the design of the studies. Nonetheless, a number of studies have shown a higher frequency of AMR in women as compared to men. In a community study from Olmsted County, 67% of patients with AMR were women whereas only 41% of patients with secondary mitral regurgitation due to left ventricular dilation and/or dysfunction (ventricular functional regurgitation) and 49% of patients with primary mitral regurgitation were women (22). In a study evaluating the etiology of mitral valve regurgitation of patients referred for surgical mitral valve repair, Glower et al observed that 78% of the patients with isolated mitral annular dilation causing significant mitral regurgitation (surrogate definition of AMR) were women (23). Additionally, a study of 378 consecutive patients with significant secondary mitral regurgitation demonstrated a higher frequency of women with AMR compared to women with ventricular functional regurgitation (64 vs. 35%, *p* < 0.001) (24). In an attempt to understand the efficacy of transcatheter edge-to-edge mitral valve repair, various studies have analyzed the subgroups of patients with AMR and have shown that the frequency of women with AMR was larger than the frequency of men with AMR (25–27).

When analyzing the frequency of AMR from the underlying pathophysiology point of view, the sex differences are less clear. A substudy of the Atherosclerosis Risk in Communities (ARIC) Study investigating the association between AF and mitral regurgitation among patients hospitalized for acute decompensated heart failure showed that among 9,104 patients with HFpEF, 2,501 had significant mitral regurgitation and 4,437 had AF. Although the mechanism of mitral regurgitation was not specified, most probably the majority of the patients had AMR since patients with primary mitral regurgitation would have been excluded as per study design. The proportion of female was comparable among patients with and without AF (67 vs. 65%, respectively) and the frequency of AF increased with increasing severity of mitral regurgitation (28). Therefore, it could be inferred that the prevalence of AMR would be higher in women than in men. In the prospective All Nippon AF In the Elderly (ANAFIE) registry, the echocardiographic substudy including 1,494 elderly patients with non-valvular AF showed that 41% of patients were female and that the proportion of significant AMR was 14%. However, it was not specified if among patients with significant AMR there were more women than men (29).

## Differences in Prevalence of ATR Between Women and Men

Several observational studies have shown that tricuspid regurgitation is more prevalent in women than in men (30–32). It was also demonstrated in a retrospective cohort study of 1,552 patients (49% of women), in which female sex, as well as age, AF, heart failure and right ventricular systolic pressure were significantly associated with tricuspid regurgitation progression (33). Specifically, the association of ATR to female sex has also been recognized (9, 34). Zhao et al analyzed factors involved in determining the severity of ATR in 170 patients with AF (56% of women), and found a female predominance in severe ATR (percentage of women 70 vs. 43% of women in non-severe ATR group, *p* < 0.001) (35). It was also observed in an echocardiographic study of 251 patients (53% of women), in which AF was strongly associated with the occurrence of significant ATR in women (OR 10.1, *p* < 0.001) but not in men (OR 0.91, *p* < 0.87) (36). This was confirmed in a non-selected population of 432 patients (49.1% of women) with AF and without primary valve disease or LV abnormalities. Significant ATR was present in 14.8% of patients, and the associated factors were female sex (OR 2.61, *p* < 0.001), LA dilation and increasing pulmonary artery systolic pressure (37). Regarding ATR in the context of HFpEF, a retrospective observational study of 328 patients detected 8% of significant ATR, with 58% of female patients among this group (11).

## Potential Causes for the Different Frequency of AMR and ATR Between Women and Men

The possible causes for the higher proportion of women with AMR and ATR as compared to men are not completely understood and remain speculative. First, some studies point to distinct factors that may lead to more advanced LA dysfunction and more atrial fibrosis in women than in men (38), higher levels of the inflammatory markers (39, 40), or different electrophysiological properties (41, 42), which may be modulated by sex hormones (43, 44). In addition, the atrioventricular annuli may have differences in composition and cellularity according to sex. In a post-mortem study, El-Busaid et al. analyzed 5-mm sections from the anterior and posterior mitral and tricuspid valve annuli and demonstrated that the myocardium was consistently present in all atrioventricular valve annuli of men but it was nearly absent in women, whereas the atrioventricular valve annuli of women were less elastic and had relatively scattered cells within the collagen matrix compared to the atrioventricular valve annuli of men (45). Insufficient compensatory leaflet remodeling in response to mitral and tricuspid annulus dilation has been demonstrated to play a role in the pathophysiology of AMR (46, 47) and ATR (48, 49). A distinct pattern of leaflet remodeling between sexes cannot be inferred from these studies yet. However, different response of fibroblasts has been demonstrated according to sex (50, 51) and could explain different prevalence of AMR and ATR between men and women. Finally, it is possible that more advanced stage of AF (18) and HFpEF (19) at the time of diagnosis and less aggressive treatment approach may also account for this greater prevalence of AMR and ATR in women.

## Clinical Perspective and Forward Thinking. A Call for Action

As a result of this higher susceptibility of women for this type of atrioventricular valve regurgitation as compared to men, strategies aimed at reducing the occurrence of AMR and ATR may have a greater impact in women than in men. The development of AMR and ATR has been associated to worse prognosis, as it entails volume overload and decreased stroke volume, but also because they are both markers of more advanced atrial remodeling due to increased AF burden, diastolic dysfunction, LA failure and pulmonary hypertension. Therefore, early diagnosis and adequate treatment of related conditions may impact on the burden of AMR and ATR, particularly in women. For instance, it is acknowledged that women with AF are diagnosed later and received less rhythm control as compared to men, as demonstrated in the EORP-AF Pilot survey (52). Subsequently, this late diagnosis and underutilization of effective therapies in women with AF may lead to a higher prevalence of significant AMR and ATR. Furthermore, successful pulmonary vein ablation for AF has been associated with lower rates of significant AMR (8) and ATR (53) during follow-up. Earlier detection of HFpEF would also be expected to reduce atrial dilation and dysfunction, and decrease the incidence of AMR. On the other side, timely diagnosis and effective treatment of pulmonary hypertension would reverse right atrial and ventricular dilation and consequently, ATR and secondary tricuspid regurgitation. Additionally, an intracavitary lead may predispose to tricuspid regurgitation (54), or worsen its severity when there are other pathogenic factors like tricuspid annular remodeling or right ventricular dilatation (55). Therefore, mode of pacing should be carefully pondered in women with high risk of tricuspid regurgitation, taking into account that leadless pacing has been also unexpectedly associated with tricuspid regurgitation progression (56). When surgery for AMR is indicated, it has been advocated to perform simultaneously a tricuspid annuloplasty in all patients to prevent future regurgitation, since right atrial enlargement is expected to continue as long as AF or HFpEF persist (57). In the setting of other left-sided valve surgery, tricuspid repair for mild or moderate tricuspid regurgitation with a dilated tricuspid annulus is advocated (class IIa) (1), and should be particularly recommended in women with additional risk factors, in order to prevent more severe forms of ATR. Furthermore, the use of surgical ablation techniques should be considered at the time of mitral valve repair/replacement in patients who are symptomatic and may be considered in patients who are asymptomatic, if feasible and if it does not increase the risk of pacemaker implantation (58). It is conceivable to hypothesize that effective rhythm control may help to halt the progression of atrial remodeling and reduce the risk of failure of mitral valve repair at long-term follow-up, although this has not been demonstrated.

The use of new percutaneous transcatheter therapies may increase in the future for the treatment of symptomatic and refractory significant AMR (25) and ATR (59), which may be preferred over valve surgery due to old age or comorbidities of these patients. In this regard, early diagnosis and treatment may be imperative in order to ensure that these therapies are effective and the patients are not referred too late when the remodeling process of the atria has reached a point of no return. Finally, echocardiographic follow-up is warranted to evaluate the development of significant AMR or ATR in patients considered at risk, for instance, those with permanent AF, HFpEF, severely dilated atria, mild-to-moderate AMR or ATR, pulmonary hypertension or those with an intracavitary lead.

There is currently a growing interest in the field of AMR and ATR due to their increasing prevalence, prognostic implications and novel therapeutic strategies. In this regard, research on sex differences in pathophysiology, clinical presentation and treatment approach is warranted and could help to better understand these heart valve diseases. On the other hand, a closer attention is required to prevent sex inequity in diagnosis and treatment of AF and HFpEF (18, 60, 61), the underlying conditions of AMR and ATR. Additionally, prompt referral to echocardiography is necessary for an earlier diagnosis, when treatment or preventive strategies may modify the natural history of these heart valve diseases.

## Conclusions

AMR and ATR occur mainly in patients with AF and HFpEF and are associated with higher rates of heart failure and mortality. Observational data demonstrate a higher prevalence of these heart valve diseases in women, especially for ATR. Mechanisms involved in this sex distribution are not well-understood, and may be related to differences in histopathological characteristics of the atrio-ventricular annuli and leaflets and different time-course or treatment strategies of the predisposing conditions in women compared to men. Research gaps include pathophysiological determinants and unbiased incidence of both heart valve diseases, as well as differential treatment strategies. Awareness of these sex-related differences from the clinical ground to the echocardiography laboratories and the investigational setting may contribute to improve the knowledge and better management of these valve disorders in both sexes.

## Author Contributions

FG-C and VD contributed to the conception and design of the paper. FG-C drafted the manuscript. JS, AB-G, and VD contributed to the critical revision of the manuscript. All authors contributed to the article and approved the submitted version.

## Conflict of Interest

VD received speaker fees from Abbott Vascular, Edwards Lifesciences, GE healthcare, Medtronic, MSD and Novartis. The remaining authors declare that the research was conducted in the absence of any commercial or financial relationships that could be construed as a potential conflict of interest.

## Publisher's Note

All claims expressed in this article are solely those of the authors and do not necessarily represent those of their affiliated organizations, or those of the publisher, the editors and the reviewers. Any product that may be evaluated in this article, or claim that may be made by its manufacturer, is not guaranteed or endorsed by the publisher.

## References

[1] Vahanian A Beyersdorf F Praz F Milojevic M Baldus S Bauersachs J. 2021 ESC/EACTS Guidelines for the management of valvular heart disease. Eur Heart J. (2021) 43:561–632. 10.1093/eurheartj/ehab626 34453165

[2] Asgar AW Mack MJ Stone GW. Secondary mitral regurgitation in heart failure: pathophysiology, prognosis, and therapeutic considerations. J Am Coll Cardiol. (2015) 65:1231–48. 10.1016/j.jacc.2015.02.009 25814231

[3] O'Gara PT Mack MJ. Secondary mitral regurgitation. N Engl J Med. (2020) 383:1458–67. 10.1056/NEJMcp1903331 33027570

[4] Benfari G Antoine C Miller WL Thapa P Topilsky Y Rossi A. Excess mortality associated with functional tricuspid regurgitation complicating heart failure with reduced ejection fraction. Circulation. (2019) 140:196–206. 10.1161/CIRCULATIONAHA.118.038946 31117814

[5] Gerçek M Rudolph V. Secondary tricuspid regurgitation: pathophysiology, incidence and prognosis. Front Cardiovasc Med. (2021) 8:701243. 10.3389/fcvm.2021.701243 34368256 PMC8339586

[6] Deferm S Bertrand PB Verbrugge FH Verhaert D Rega F Thomas JD. Atrial functional mitral regurgitation: JACC review topic of the week. J Am Coll Cardiol. (2019) 73:2465–76. 10.1016/j.jacc.2019.02.061 31097168

[7] Silbiger JJ. Atrial functional tricuspid regurgitation: an underappreciated cause of secondary tricuspid regurgitation. Echocardiography. (2019) 36:954–7. 10.1111/echo.14327 30919501

[8] Gertz ZM Raina A Saghy L Zado ES Callans DJ Marchlinski FE. Evidence of atrial functional mitral regurgitation due to atrial fibrillation: reversal with arrhythmia control. J Am Coll Cardiol. (2011) 58:1474–81. 10.1016/j.jacc.2011.06.032 21939832

[9] Utsunomiya H Itabashi Y Mihara H Berdejo J Kobayashi S Siegel RJ. Functional tricuspid regurgitation caused by chronic atrial fibrillation: a real-time 3-dimensional transesophageal echocardiography study. Circ Cardiovasc Imaging. (2017) 10:e004897. 10.1161/CIRCIMAGING.116.004897 28073806

[10] Tamargo M Obokata M Reddy YNV Pislaru SV Lin G Egbe AC. Functional mitral regurgitation and left atrial myopathy in heart failure with preserved ejection fraction. Eur J Heart Fail. (2020) 22:489–98. 10.1002/ejhf.1699 31908127

[11] Harada T Obokata M Omote K Iwano H Ikoma T Okada K. Functional tricuspid regurgitation and right atrial remodeling in heart failure with preserved ejection fraction. Am J Cardiol. (2022) 162:129–35. 10.1016/j.amjcard.2021.09.021 34702555

[12] Bisbal F Baranchuk A Braunwald E Bayés de Luna A Bayés-Genís A. Atrial failure as a clinical entity. J Am Coll Cardiol. (2020) 75:222–32. 10.1016/j.jacc.2019.11.013 31948652

[13] Omote K Borlaug BA. Left atrial myopathy in heart failure with preserved ejection fraction. Circ J. (2021). 10.1253/circj.CJ-21-0795 34645733

[14] Abe Y Akamatsu K Ito K Matsumura Y Shimeno K Naruko T. Prevalence and prognostic significance of functional mitral and tricuspid regurgitation despite preserved left ventricular ejection fraction in atrial fibrillation patients. Circ J. (2018) 82:1451–8. 10.1253/circj.CJ-17-1334 29553091

[15] Saito C Minami Y Arai K Haruki S Yagishita Y Jujo K. Prevalence, clinical characteristics, and outcome of atrial functional mitral regurgitation in hospitalized heart failure patients with atrial fibrillation. J Cardiol. (2018) 72:292–9. 10.1016/j.jjcc.2018.04.002 29752195

[16] Prapan N Ratanasit N Karaketklang K. Significant functional tricuspid regurgitation portends poor outcomes in patients with atrial fibrillation and preserved left ventricular ejection fraction. BMC Cardiovasc Disord. (2020) 20:433. 10.1186/s12872-020-01716-6 33023481 PMC7541233

[17] Wang TKM Akyuz K Mentias A Kirincich J Duran Crane A Xu S. contemporary etiologies, outcomes, and novel risk score for isolated tricuspid regurgitation. JACC Cardiovasc Imaging. (2021) 15:731–44. 10.1016/j.jcmg.2021.10.015 34922866

[18] Ko D Rahman F Schnabel RB Yin X Benjamin EJ Christophersen IE. Atrial fibrillation in women: epidemiology, pathophysiology, presentation, and prognosis. Nat Rev Cardiol. (2016) 13:321–32. 10.1038/nrcardio.2016.45 27053455 PMC5579870

[19] Dewan P Rørth R Raparelli V Campbell RT Shen L Jhund PS. Sex-related differences in heart failure with preserved ejection fraction. Circ Heart Fail. (2019) 12:e006539 10.1161/CIRCHEARTFAILURE.119.006539 31813280

[20] Vakamudi S Jellis C Mick S Wu Y Gillinov AM Mihaljevic T. Sex differences in the etiology of surgical mitral valve disease. Circulation. (2018) 138:1749–51. 10.1161/CIRCULATIONAHA.118.035789 30354470

[21] Martínez-Sellés M García-Fernández MA Moreno M Larios E García-Robles JA Pinto A. Influence of gender on the etiology of mitral regurgitation. Rev Esp Cardiol. (2006) 59:1335–8. 10.1016/S1885-5857(07)60091-7 17194432

[22] Dziadzko V Dziadzko M Medina-Inojosa JR Benfari G Michelena HI Crestanello JA. Causes and mechanisms of isolated mitral regurgitation in the community: clinical context and outcome. Eur Heart J. (2019) 40:2194–202. 10.1093/eurheartj/ehz314 31121021

[23] Glower DD Bashore TM Harrison JK Wang A Gehrig T Rankin JS. Pure annular dilation as a cause of mitral regurgitation: a clinically distinct entity of female heart disease. J Heart Valve Dis. (2009) 18:284–8. 19557984

[24] Okamoto C Okada A Nishimura K Moriuchi K Amano M Takahama H. Prognostic comparison of atrial and ventricular functional mitral regurgitation. Open Heart. (2021) 8:e001574. 10.1136/openhrt-2021-001574 33589540 PMC7887352

[25] Popolo Rubbio A Testa L Grasso C Sisinni A Tusa M Agricola E. Transcatheter edge-to-edge mitral valve repair in atrial functional mitral regurgitation: insights from the multi-center MITRA-TUNE registry. Int J Cardiol. (2022) 349: 39–45 10.1016/j.ijcard.2021.11.027 34826500

[26] Claeys MJ Debonnaire P Bracke V Bilotta G Shkarpa N Vanderheyden M. Clinical and hemodynamic effects of percutaneous edge-to-edge mitral valve repair in atrial vs. ventricular functional mitral regurgitation. Am J Cardiol. (2021) 161:70–5. 10.1016/j.amjcard.2021.08.062 34794621

[27] Benito-González T Carrasco-Chinchilla F Estévez-Loureiro R Pascual I Arzamendi D Garrote-Coloma C. Clinical and echocardiographic outcomes of transcatheter mitral valve repair in atrial functional mitral regurgitation. Int J Cardiol. (2021) 345:29–35. 10.1016/j.ijcard.2021.09.056 34610357

[28] Arora S Brown ZD Sivaraj K Hendrickson MJ Mazzella AJ Chang PP. The relationship between atrial fibrillation, mitral regurgitation, and heart failure subtype: the ARIC study. J Card Fail. (2021). 10.1016/j.cardfail.2021.10.015. [Epub ahead of print]. 34955335

[29] Hiasa KI Kaku H Kawahara G Inoue H Yamashita T Akao M. Echocardiographic structure and function in elderly patients with atrial fibrillation in Japan-The ANAFIE echocardiographic substudy. Circ J. (2022) 86:222–32. 10.1253/circj.CJ-21-0180 34937815

[30] Ong K Yu G Jue J. Prevalence and spectrum of conditions associated with severe tricuspid regurgitation. Echocardiography. (2014) 31:558–62. 10.1111/echo.12420 24304325

[31] Singh JP Evans JC Levy D Larson MG Freed LA Fuller DL. Prevalence and clinical determinants of mitral, tricuspid, and aortic regurgitation (the Framingham Heart Study). Am J Cardiol. (1999) 83:897–902. 10.1016/S0002-9149(98)01064-9 10190406

[32] Mutlak D Aronson D Lessick J Reisner SA Dabbah S Agmon Y. Functional tricuspid regurgitation in patients with pulmonary hypertension: is pulmonary artery pressure the only determinant of regurgitation severity? Chest. (2009) 135:115–21. 10.1378/chest.08-0277 18719061

[33] Mutlak D Khalil J Lessick J Kehat I Agmon Y Aronson D. risk factors for the development of functional tricuspid regurgitation and their population-attributable fractions. JACC Cardiovasc Imaging. (2020) 13:1643–51. 10.1016/j.jcmg.2020.01.015 32305485

[34] Topilsky Y Nkomo VT Vatury O Michelena HI Letourneau T Suri RM. Clinical outcome of isolated tricuspid regurgitation. JACC Cardiovasc Imaging. (2014) 7:1185–94. 10.1016/j.jcmg.2014.07.018 25440592

[35] Zhao SX Soltanzad N Swaminathan A Ogden WD Schiller NB. Frequency and associated clinical features of functional tricuspid regurgitation in patients with chronic atrial fibrillation. Am J Cardiol. (2017) 119: 1371–7 10.1016/j.amjcard.2017.01.037 28284370

[36] Gual-Capllonch F Cediel G Ferrer E Teis A Juncà G Vallejo N. Sex-related differences in the mechanism of functional tricuspid regurgitation. Heart Lung Circ. (2021) 30:e16–22. 10.1016/j.hlc.2020.06.018 32771383

[37] Gual-Capllonch F Cediel G Teis A Ferrer-Sistach E Borrellas A Juncà G. Prevalence and factors associated with atrial mitral and tricuspid regurgitation in patients with atrial fibrillation. Echocardiography. (2021) 38:2043–51. 10.1111/echo.15257 34845760

[38] Cochet H Mouries A Nivet H Sacher F Derval N Denis A. Age, atrial fibrillation, and structural heart disease are the main determinants of left atrial fibrosis detected by delayed-enhanced magnetic resonance imaging in a general cardiology population. J Cardiovasc Electrophysiol. (2015) 26:484–92. 10.1111/jce.12651 25727248

[39] Khera A McGuire DK Murphy SA Stanek HG Das SR Vongpatanasin W. Race and gender differences in C-reactive protein levels. J Am Coll Cardiol. (2005) 46:464–9. 10.1016/j.jacc.2005.04.051 16053959

[40] Ix JH Katz R Kestenbaum BR de Boer IH Chonchol M Mukamal KJ. Fibroblast growth factor-23 and death, heart failure, and cardiovascular events in community-living individuals: CHS (Cardiovascular Health Study). J Am Coll Cardiol. (2012) 60:200–7. 10.1016/j.jacc.2012.03.040 22703926 PMC3396791

[41] Takigawa M Kuwahara T Takahashi A Watari Y Okubo K Takahashi Y. Differences in catheter ablation of paroxysmal atrial fibrillation between males and females. Int J Cardiol. (2013) 168:1984–91. 10.1016/j.ijcard.2012.12.101 23782910

[42] Kim JS Shin SY Kang JH Yong HS Na JO Choi CU. influence of sex on the association between epicardial adipose tissue and left atrial transport function in patients with atrial fibrillation: a multislice computed tomography study. J Am Heart Assoc. (2017) 6:e006077. 10.1161/JAHA.117.006077 28778939 PMC5586448

[43] Yang PC Kurokawa J Furukawa T Clancy CE. Acute effects of sex steroid hormones on susceptibility to cardiac arrhythmias: a simulation study. PLoS Comput Biol. (2010) 6:e1000658. 10.1371/journal.pcbi.1000658 20126530 PMC2813260

[44] Tsai WC Chen YC Lin YK Chen SA Chen YJ. Sex differences in the electrophysiological characteristics of pulmonary veins and left atrium and their clinical implication in atrial fibrillation. Circ Arrhythm Electrophysiol. (2011) 4:550–9. 10.1161/CIRCEP.111.961995 21659634

[45] El-Busaid H Hassan S Odula P. Ogeng'o J, Ndung'u B. Sex variations in the structure of human atrioventricular annuli. Folia Morphol. (2012) 71:23–7. 22532181

[46] Kim DH Heo R Handschumacher MD Lee S Choi YS Kim KR. Mitral valve adaptation to isolated annular dilation: insights into the mechanism of atrial functional mitral regurgitation. JACC Cardiovasc Imaging. (2019) 12:665–77. 10.1016/j.jcmg.2017.09.013 29248661 PMC5993562

[47] Kagiyama N Hayashida A Toki M Fukuda S Ohara M Hirohata A. Insufficient leaflet remodeling in patients with atrial fibrillation: association with the severity of mitral regurgitation. Circ Cardiovasc Imaging. (2017) 10:e005451. 10.1161/CIRCIMAGING.116.005451 28289019

[48] Afilalo J Grapsa J Nihoyannopoulos P Beaudoin J Gibbs JS Channick RN. Leaflet area as a determinant of tricuspid regurgitation severity in patients with pulmonary hypertension. Circ Cardiovasc Imaging. (2015) 8:e002714. 10.1161/CIRCIMAGING.114.002714 25977303 PMC4435735

[49] Van Rosendael PJ Joyce E Katsanos S Debonnaire P Kamperidis V van der Kley F. Tricuspid valve remodelling in functional tricuspid regurgitation: multidetector row computed tomography insights. Eur Heart J Cardiovasc Imaging. (2016) 17:96–105. 10.1093/ehjci/jev140 26060205

[50] Dworatzek E Mahmoodzadeh S Schriever C Kusumoto K Kramer L Santos G. Sex-specific regulation of collagen I and III expression by 17β-Estradiol in cardiac fibroblasts: role of estrogen receptors. Cardiovasc Res. (2019) 115:315–27. 10.1093/cvr/cvy185 30016401 PMC6933535

[51] Walker CJ Schroeder ME Aguado BA Anseth KS Leinwand LA. Matters of the heart: cellular sex differences. J Mol Cell Cardiol. (2021) 160:42–55. 10.1016/j.yjmcc.2021.04.010 34166708 PMC8571046

[52] Lip GY Laroche C Boriani G Cimaglia P Dan GA Santini M. Sex-related differences in presentation, treatment, and outcome of patients with atrial fibrillation in Europe: a report from the Euro Observational Research Programme Pilot survey on Atrial Fibrillation. Europace. (2015) 17:24–31. 10.1093/europace/euu155 24957921

[53] Markman TM Plappert T De Feria Alsina A Levin M Amankwah N Sheth S. Improvement in tricuspid regurgitation following catheter ablation of atrial fibrillation. J Cardiovasc Electrophysiol. (2020) 31:2883–8. 10.1111/jce.14707 32757450

[54] Chang JD Manning WJ Ebrille E Zimetbaum PJ. Tricuspid valve dysfunction following pacemaker or cardioverter-defibrillator implantation. J Am Coll Cardiol. (2017) 69:2331–41. 10.1016/j.jacc.2017.02.055 28473139

[55] Riesenhuber M Spannbauer A Gwechenberger M Pezawas T Schukro C Stix G. Pacemaker lead-associated tricuspid regurgitation in patients with or without pre-existing right ventricular dilatation. Clin Res Cardiol. (2021) 110:884–94. 10.1007/s00392-021-01812-3 33566185 PMC8166708

[56] Beurskens NEG Tjong FVY de Bruin-Bon RHA Dasselaar KJ Kuijt WJ Wilde AAM. Impact of leadless pacemaker therapy on cardiac and atrioventricular valve function through 12 months of follow-up. Circ Arrhythm Electrophysiol. (2019) 12:e007124. 10.1161/CIRCEP.118.007124 31060371

[57] Shibata T Takahashi Y Fujii H Morisaki A Abe Y. Surgical considerations for atrial functional regurgitation of the mitral and tricuspid valves based on the etiological mechanism. Gen Thorac Cardiovasc Surg. (2021) 69:1041–9. 10.1007/s11748-021-01629-x 33970433 PMC8203518

[58] Gillinov AM Gelijns AC Parides MK DeRose JJ Jr Moskowitz AJ Voisine P. Surgical ablation of atrial fibrillation during mitral-valve surgery. N Engl J Med. (2015) 372:1399–409. 10.1056/NEJMoa1500528 25853744 PMC4664179

[59] Lurz P Stephan von Bardeleben R Weber M Sitges M Sorajja P Hausleiter J. Transcatheter Edge-to-Edge Repair for Treatment of Tricuspid Regurgitation. J Am Coll Cardiol. (2021) 77:229–39. 10.1016/j.jacc.2020.11.038 33478646

[60] Sotomi Y Hikoso S Nakatani D Mizuno H Okada K Dohi T. Sex differences in heart failure with preserved ejection fraction. J Am Heart Assoc. (2021) 10:e018574. 10.1161/JAHA.120.018574 33619973 PMC8174270

[61] Farrero M Bellumkonda L Gómez Otero I Díaz Molina B. Sex and heart failure treatment prescription and adherence. Front Cardiovasc Med. (2021) 8:630141. 10.3389/fcvm.2021.630141 34026865 PMC8137967

